# Investigation on urinary and serum alpha klotho in dogs with chronic kidney disease

**DOI:** 10.1186/s12917-020-02458-5

**Published:** 2020-07-16

**Authors:** Hong jae Yi, Jong bok Lee, Kyu pil Lee, Ye-In Oh, Kun ho Song, Kyoung won Seo

**Affiliations:** 1 VMTH of College of Veterinary Medicine, Chungnam National University, N13-2, #308, 99 Daehak-ro, Yuseong-gu, Daejeon, Republic of Korea; 2 College of Veterinary Medicine, Kangwon National University, Chuncheon, Korea

**Keywords:** Canine, Chronic kidney disease-mineral bone disorder (CKD-MBD), Klotho, SDMA, Serum, Urine

## Abstract

**Background:**

As a co-receptor for fibroblast growth factor 23, klotho plays a pivotal role in phosphate metabolism. The kidney is known to be the main source of soluble alpha-klotho and the principal regulator of its concentration. Previous studies in human participants showed that the concentration of soluble alpha-klotho in serum and urine decreased in chronic kidney disease (CKD) patients. However, no previous study has assessed soluble alpha-klotho levels in dogs. This study aimed to measure serum and urinary alpha-klotho levels in CKD dogs and identify their associations with International Renal Interest Society (IRIS) CKD stages and other parameters known to be associated with CKD.

**Results:**

Serum and urinary alpha klotho concentrations were measured by a commercially available canine-specific sandwich enzyme-linked immunosorbent assay kit and compared between groups by a nonparametric Kruskal–Wallis test. Spearman’s correlation coefficient was used to evaluate the relationships between variables. A stepwise multiple regression analysis was performed to estimate the effects of independent predictors on klotho concentrations. The urine klotho-to-creatinine ratio (UrKl/Cr) was significantly lower in stage 3 dogs than the control group and was significantly lower in dogs with stage 3 and 4 CKD than in those with stage 1 and 2 disease. UrKl/Cr was negatively correlated with serum symmetric dimethylarginine (sSDMA), blood urea nitrogen (BUN), creatinine, and phosphorus concentration. Serum alpha-klotho concentration in dogs with stages 2 and 3 CKD was significantly lower than those in the control group. There was no significant correlation between serum alpha-klotho and BUN, creatinine, and phosphorus concentrations. No statistically significant differences were observed in UrKl/Cr and serum alpha-klotho concentration between groups based on sex, age, urine protein-to-creatinine ratio (UPC), or blood pressure.

**Conclusions:**

UrKl/Cr decreased in dogs with advanced CKD, and it was negatively correlated with sSDMA, BUN, creatinine, and phosphorus concentrations. Thus, klotho is associated with CKD and its clinical consequences, including CKD-mineral bone disorder, in dogs. Although serum klotho concentration was negatively correlated with sSDMA levels, it was not apparently related to IRIS CKD stage or other parameters known to be associated with CKD.

## Background

Chronic kidney disease (CKD) is one of the most common kidney diseases in dogs. The prevalence of CKD in dogs was estimated to be 1.5% in general practice [1]. Reduced glomerular filtration rate (GFR) can decrease phosphorus excretion and result in hyperphosphatemia. Renal secondary hyperparathyroidism (RSHP), which is generally believed to be the clinical consequence of hyperphosphatemia, is reported in around 76% of CKD dogs, and in over 96% of dogs with over International Renal Interest Society (IRIS) stage 3 and over of the disease [2, 3]. The term “chronic kidney disease-mineral bone disorder” (CKD-MBD) has been used in humans and animals to describe a condition characterized by renal osteodystrophy and abnormal mineral metabolism with high levels of serum phosphate, fibroblast growth factor (FGF)-23, and parathyroid hormone (PTH) [4–6]. Klotho deficiency was suggested to be associated with or even induce these abnormal metabolic consequences [7–11].

The *klotho* gene was accidentally discovered in mice in 1997 [12]. Two forms of klotho protein, membranous and soluble, are consistently detected in vivo. The membranous form is mainly expressed on the proximal and distal tubules of the kidney [12–14]. Interactions of FGF-23 and the FGF receptor and subsequent signaling require binding of membranous klotho to FGF receptors and formation of the klotho-FGF receptor complex, which binds to FGF-23 with much higher affinity than klotho or the FGF receptor alone [15–17]. Under physiological conditions, FGF-23 suppresses PTH and calcitriol secretion to reduce serum phosphorus concentrations. In CKD, decreased expression of membrane-bound klotho limits FGF-23-mediated signal transduction through FGF receptor-klotho complexes, resulting in an increase in parathyroid hormone levels [5]. Moreover, soluble klotho has been reported to directly control phosphorus excretion in the kidney and participate in systemic mineral homeostasis by regulating 1α-hydroxylase activity and PTH and FGF-23 secretion [18–20].

Soluble alpha-klotho is generated when the extracellular domain of membranous alpha-klotho is cleaved and released into plasma [21–24]. Soluble alpha-klotho is a major functional form in the circulation [25, 26] and is also found in cerebrospinal fluid and urine [27–29]. The kidney is known to be the main source of soluble alpha-klotho and the principal regulator of its concentration [24, 30, 31]. Therefore, it seems reasonable to assume that nephron loss can lead to reduced production and release of soluble klotho. Significantly reduced levels of renal alpha-klotho mRNA were observed in several studies with rodent CKD models [31, 32]. Human studies have demonstrated that the soluble alpha-klotho concentration in serum and urine is decreased in CKD patients [27, 33] and that the serum alpha-klotho level tends to reduce as the CKD stage advances [34]. However, in contrast, some studies have reported no change in klotho concentration depending on the kidney function [35–37].

Recently, one study confirmed that the concentration of plasma FGF-23 increases as CKD advances and is significantly different between IRIS stages 1 and 2 versus stages 3 and 4 in dogs [38]. However, to the authors' knowledge, no previous study has assessed soluble alpha-klotho, which plays a key role with FGF-23 in CKD-MBD in dogs. Therefore, the aim of this study was to identify the characteristics of soluble alpha-klotho in CKD dogs by measuring serum and urinary alpha-klotho levels and verify their association with IRIS CKD stages and other parameters, including serum SDMA (sSDMA), creatinine, blood urea nitrogen (BUN), and phosphorus concentrations, which are known to be associated with CKD.

## Results

**Fig. 1:**
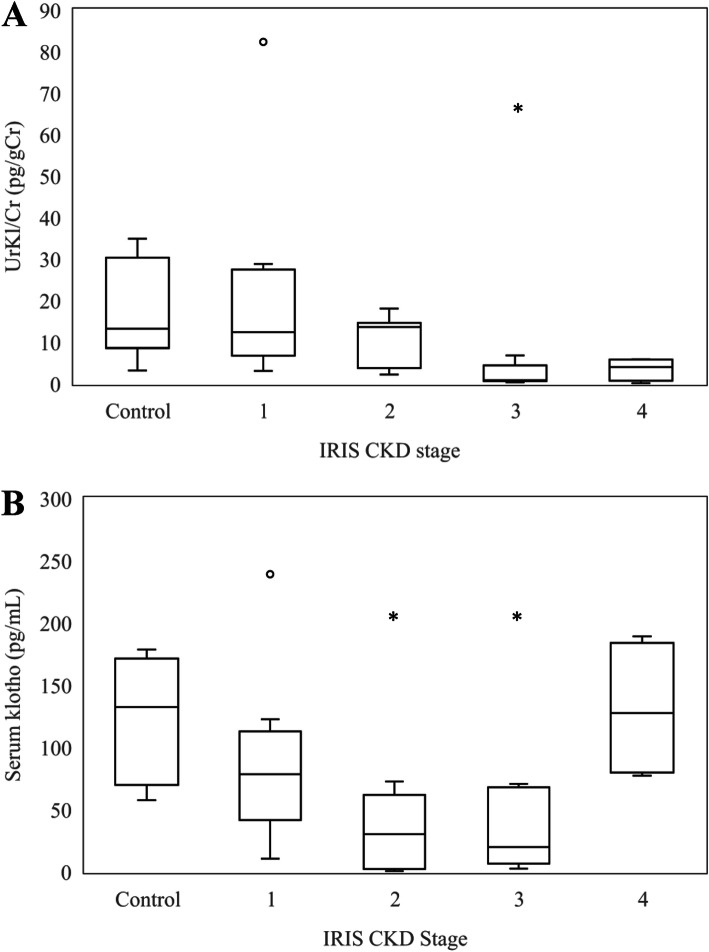
Box-and-whisker plots of urinary (**a**) and serum (**b**) klotho concentrations. The boxes represent the 25th and 75thpercentiles, and the central lines in the boxes represent the median values.The whiskers represent the range of concentrations. Dots represent outliers. **a** The asterisk represents a statistically lower UrKl/Cr in dogs with IRISstages 3 (*p* = 0.001) compared to control dogs. **b** Asterisk represents astatistically lower serum klotho concentration in dogs with IRIS stages 2 (*p*= 0.001) and 3 (*p* = 0.001) compared to control dogs.

**Fig. 2:**
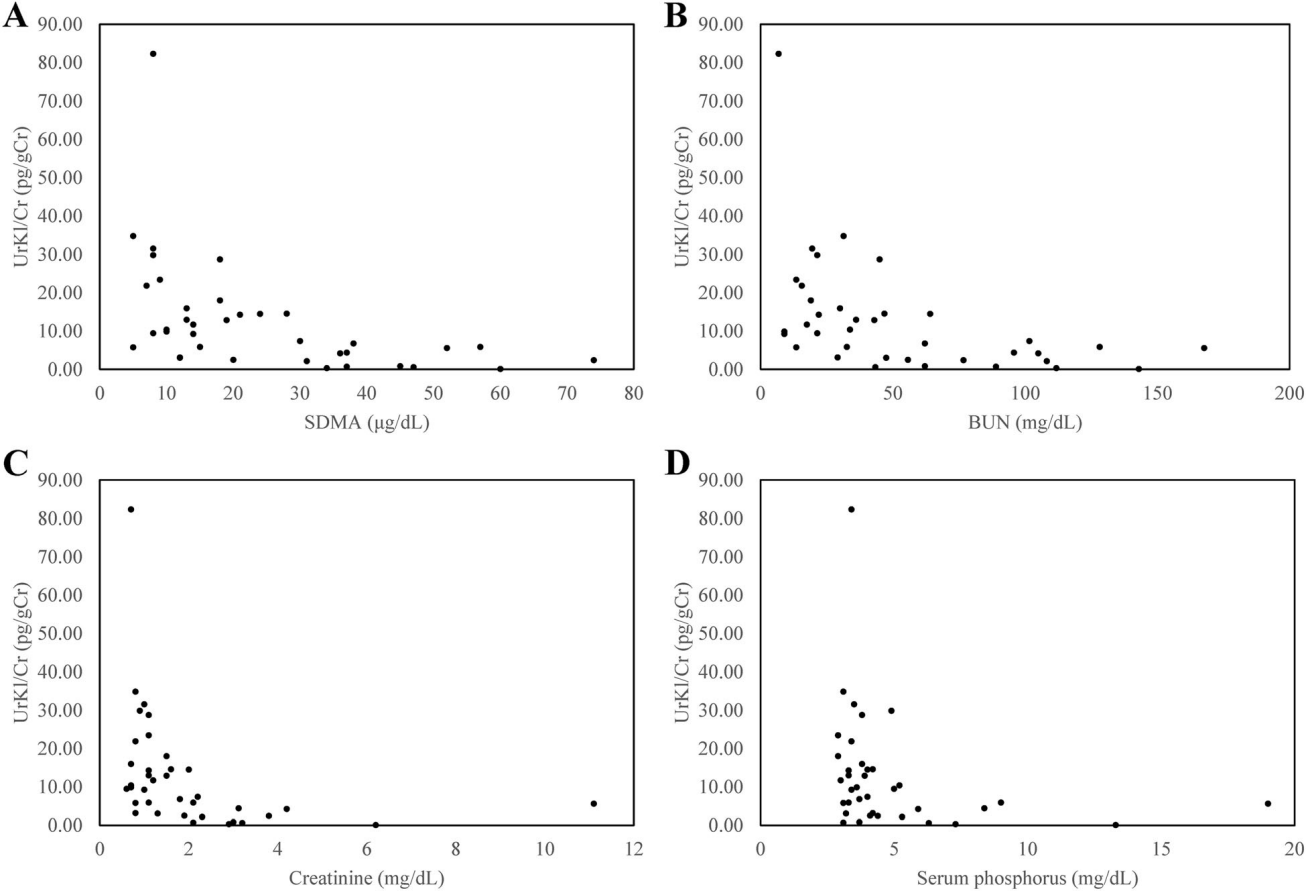
Relationships between UrKl/Cr and **a** serum SDMA, **b** BUN, **c** creatinine, and**d** phosphorus concentrations. UrKl/Crwas negatively correlated with sSDMA (*r* = -0.661, *p* < 0.001),BUN (*r* = -0.631, *p* < 0.001), creatinine (*r* = -0.651, *p*< 0.001) and phosphorus (*r* = -0.449, *p* = 0.005) concentrations.Spearman’s correlation coefficient was used to evaluate the relationshipsbetween variables.

The median age of dogs in the CKD group and control group were significantly different (*p* < 0.001). The results of laboratory analysis measuring the urine klotho-to-creatinine ratio (UrKl/Cr) and the serum alpha-klotho, sSDMA, BUN, creatinine, and phosphorus levels are presented in Table 1. The soluble alpha-klotho analysis was performed with serum and urine samples. Calculated UrKl/Cr was used to minimize the effect of urine specific gravity. The median UrKl/Cr (range) for all CKD dogs was lower (5.85 pg/gCr, 0.08 to 82.28) than that of control dogs (13.16 pg/gCr, 5.75 to 34.84). UrKl/Cr was significantly lower in stage 3 dogs than the control group (*p* = 0.001) and stage 1 CKD group (*p* = 0.003; Fig. 1). Serum alpha-klotho concentration was significantly lower in dogs with stage 2 (*p* = 0.001) and 3 (*p* = 0.001) CKD in comparison with the control group. UrKl/Cr in dogs with stage 3 and 4 CKD was significantly lower than that in dogs with stages 1 and 2 CKD (*p* < 0.001). UrKl/Cr was negatively correlated with sSDMA (*r* = -0.661, *p* < 0.001; Fig. 2), BUN (*r* = -0.631, *p* < 0.001; Fig. 2), creatinine (*r* = -0.651, *p* < 0.001; Fig. 2), and phosphorus (*r* = -0.449, *p* = 0.005; Fig. 2) concentrations.

**Table 1:** Comparison of laboratory variables in control and chronic kidney disease dogs

Laboratory Variable	Control (*n* = 10)	All CKD (*n* = 27)	Stage 1 (*n* = 8)	Stage 2 (*n* = 8)	Stage 3 (*n* = 7)	Stage 4 (*n* = 4)
UrKl/Cr (pg/gCr)	13.16 (5.75–34.84)	5.85 (0.08–82.28)	12.32 (3.03–82.28)	13.57 (2.15-18.00)	0.81 (0.30–4.37)	3.98 (0.08–5.85)
Serum Klotho (pg/mL)	132.25 (57.46-169.33)	59.13 (0.47-189.13)	78.32 (10.47-122.47)	30.14 (0.47–72.47)	19.8 (2.47–70.47)	127.47 (77.13-189.13)
SDMA (µg/dL)	8 (5–13)	28 (8–74)	13.5 (8–17)	22.5 (18–31)	37 (34–47)	58.5 (52–74)
Creatinine (mg/dL)	0.8 (0.6-1)	1.9 (0.7–11.1)	1.1 (0.7–1.3)	1.75 (1.5–2.3)	3 (1.8–4.2)	5 (3.8–11.1)
BUN (mg/dL)	21.4 (9-33.8)	55.7 (6.8-167.7)	25.05 (6.8–47.5)	51.25 (19-108.2)	89 (43.4-111.8)	135.6 (76.7-167.7)
Phosphorus (mg/dL)	3.7 (3.1–5.2)	3.9 (2.9–19)	3.3 (2.9–3.8)	4 (2.9–5.3)	5.9 (3.1–8.38)	11.15 (4.39-19)

Results are presented as median and range.

UrKl/Cr, urine klotho-to-creatinine ratio; SDMA, symmetric dimethylarginine; BUN, blood urea nitrogen.

**Fig. 3:**
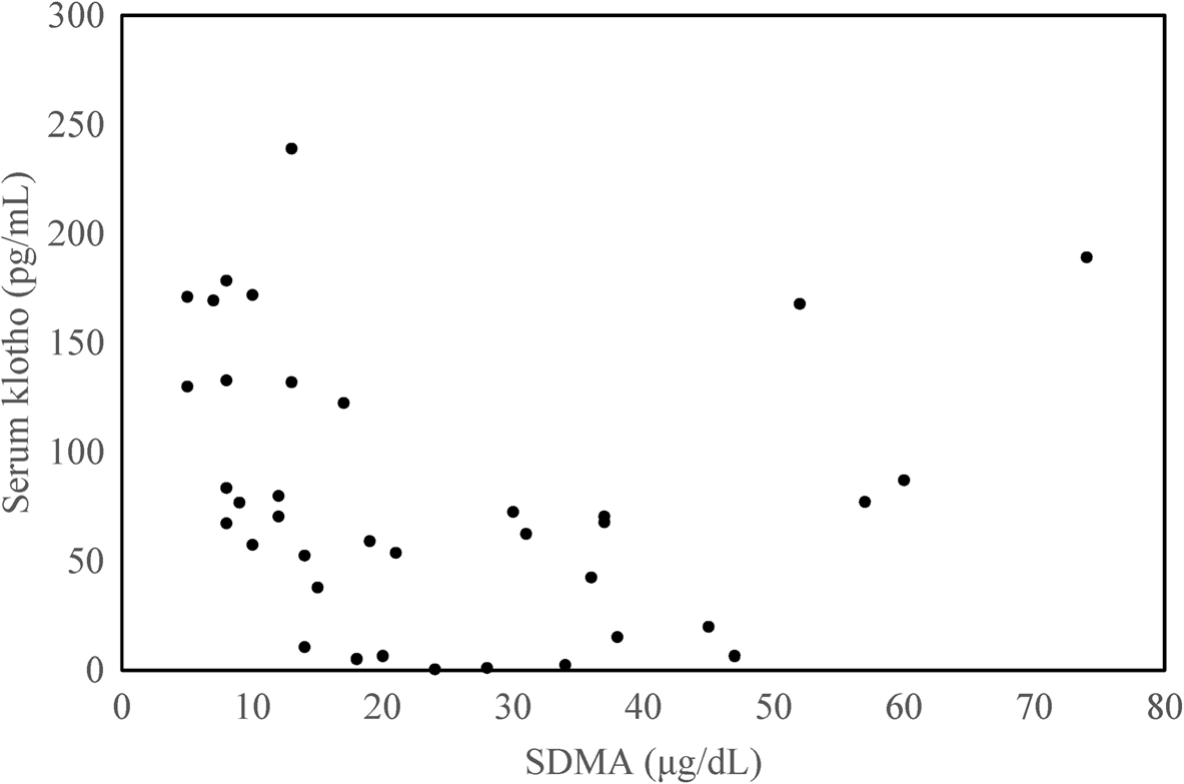
Relationship between serum alpha-klotho andsSDMA concentrations. Serum alpha klotho was negatively correlated with sSDMAconcentration (*r* = -0.348, *p* = 0.035).

**Fig. 4:**
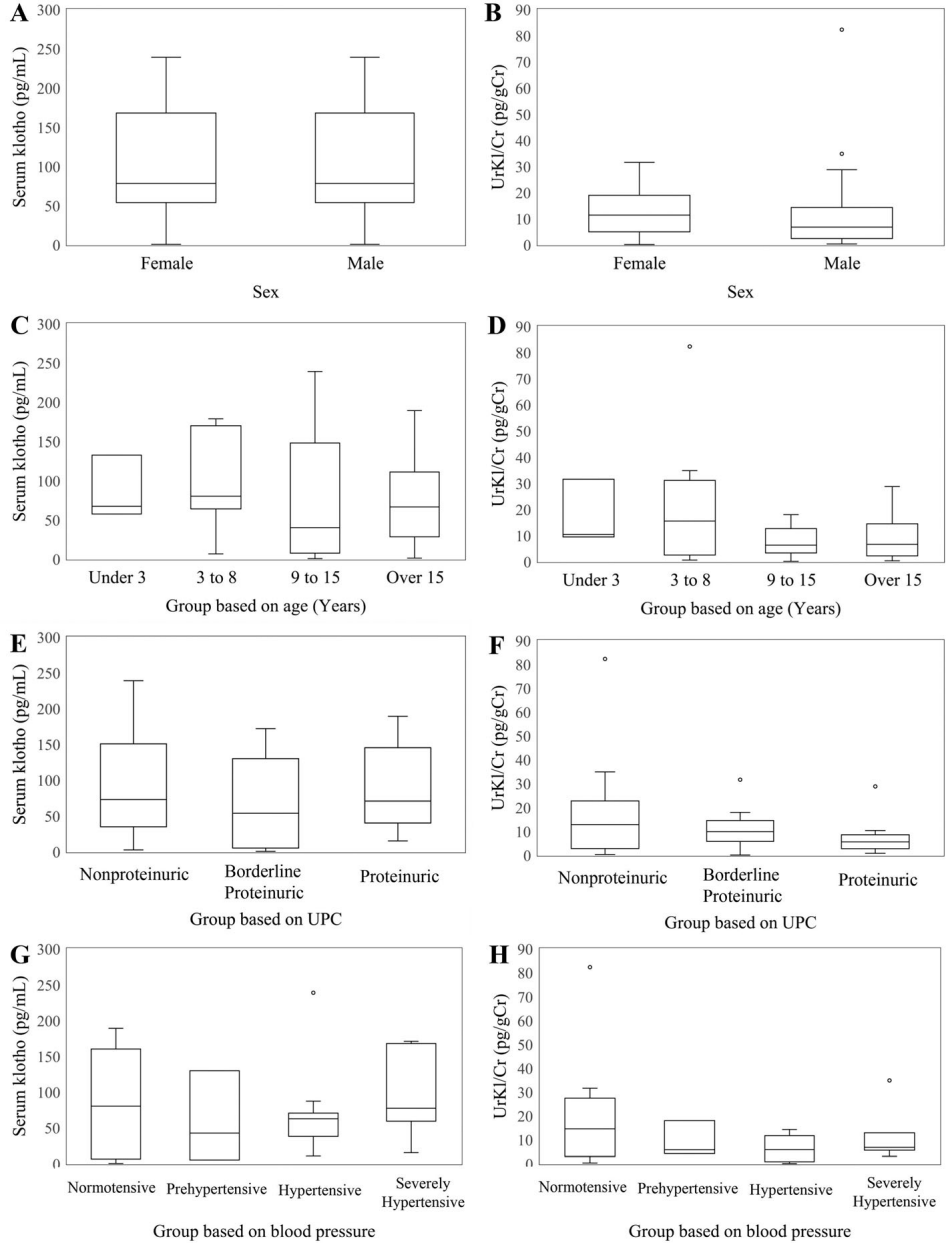
Box-and-whisker plots illustrating klothoconcentrations based on sex, age, UPC, and blood pressure. The boxes representthe 25th and 75th percentiles, and the central lines in the boxes represent themedian values. The whiskers represent the range of concentrations. Dotsrepresent outliers. Serum klotho and sex (**a**),age (**c**), UPC (**e**), and blood pressure (**g**).Urinary klotho-to-creatinine ratio (UrKl/Cr) and sex (**b**), age (**d**), UPC (**f**), and blood pressure (**h**).

The median(range) serum alpha-klotho level in all CKD dogs was lower (59.13pg/mL, 0.47 to 189.13 pg/mL) than that in control dogs (132.25 pg/mL, 57.46 to169.33 pg/mL; Table 1). Serum alpha-klotho concentration was negativelycorrelated with sSDMA level (*r* = -0.348, *p* = 0.035; Figure 3).There was no significant correlation between serum alpha-klothoconcentration and BUN (*p* = 0.297), creatinine (*p* = 0.061), andphosphorus (*p* = 0.693) concentration. There was no statisticallysignificant difference in UrKl/Cr and serum alpha klothoconcentration between different groups based on sex, age, urineprotein-to-creatinine ratio (UPC), or blood pressure (Figure 4).

Stepwise multiple regressionanalysis was performed with sSDMA, BUN, creatinine, and phosphorusconcentrations to predict UrKl/Cr. Of these variables, only sSDMA concentration(ß = -0.479) wasidentified as a predictive variable (total R^2^ = 0.229, *p* =0.003).

## Discussion

To the authors’ knowledge, this is the first study assessing soluble klotho in dogs. In the current study, the characteristics of serum and urinary klotho were investigated in CKD dogs. UrKl/Cr was significantly lower in stage 3 CKD group in comparison with those in the control and stage 1 groups. In addition, UrKl/Cr of stage 3 and stage 4 CKD dogs was significantly lower than that of stage 1 and stage 2 CKD dogs. Median UrKl/Cr in the stage 4 CKD group was lower than that in the early stages, although it was not significantly different from that in the control group. Moreover, UrKl/Cr was inversely correlated with all tested parameters, including sSDMA, creatinine, BUN, and phosphorus levels, in CKD dogs. In addition, stepwise multiple regression analysis showed that sSDMA was an independent predictor of UrKl/Cr. The significantly lower UrKl/Cr in dogs with progressed CKD and its correlation with various parameters reflecting either kidney function or the severity of kidney disease suggests that klotho is associated with CKD and the development of its clinical consequences in dogs.

In a previous study on dogs with CKD, increased levels of FGF-23 and PTH were confirmed in IRIS CKD stage 3 and 4 dogs [38]. However, measurable concentrations of klotho could not be detected in that study using the alpha-klotho enzyme-linked immunosorbent assay (ELISA) kit of IBL-America (Minneapolis, USA), which was the only available ELISA kit for detecting human or mouse klotho protein. The alpha-klotho protein sequence is known to be 88% identical between humans and dogs and 98% identical between humans and mice [39]. Since using the ELISA kit manufactured especially for canine tests was expected to enhance the accuracy of the test, the ELISA kit measuring canine-specific soluble alpha klotho was used in this study. Additionally, serum and urine samples were never thawed before ELISA assay because a recent study showed that the ELISA value of serum klotho decreases as samples undergo repeated freeze-thaw cycles [40].

Notably, the UrKl/Cr of dogs with advanced CKD was significantly reduced and correlated with various parameters, while serum alpha-klotho did not show these correlations in our study. This finding suggests that urinary klotho is more associated with kidney function or damage than serum klotho. It is also consistent with the reported characteristics of soluble klotho in CKD patients, which indicated that the amount of urinary klotho, rather than serum klotho, are linked to the extent of nephron function [27]. In this study, the amount of urinary klotho excreted for 24 h trended towards lower values as the CKD stage advanced, whereas serum klotho levels did not. The correlations with various parameters related to renal function and significant differences between advanced stages and early stages suggest that UrKl/Cr has potential as a biomarker of kidney disease or can be used when monitoring disease progression. A possible advantage of using urinary klotho as a diagnostic or monitoring tool is that it is noninvasive in comparison with existing tools requiring venipuncture. Further studies are needed to confirm its usefulness as a biomarker of kidney disease.

Contrary to UrKl/Cr, serum alpha-klotho concentration in stage 4 CKD dogs was higher than that in stage 3 CKD dogs, although the concentrations in stage 2 and stage 3 CKD dogs were significantly lower than those in control dogs. Interestingly, the relationship between serum klotho and sSDMA was better explained by a quadratic model (*p* < 0.001, R^2^ = 0.407) than a linear model (*p* = 0.428, R^2^ = 0.018). To date, no published studies of klotho have suggested a biological explanation related to this phenomenon. Further study is needed to validate this phenomenon and clarify the biological mechanisms underpinning it. Among the parameters examined, only sSDMA was negatively associated with serum alpha-klotho concentration. These results seem to correspond with the findings of human studies showing that plasma levels of serum klotho were not related to kidney function [37]. In a large cohort study of 312 patients, plasma klotho levels did not differ across CKD stages and were not significantly associated with GFR or other parameters of calcium-phosphate metabolism, which contradicted previous studies in which serum alpha-klotho concentration was associated with estimated GFR (eGFR) and was significantly decreased in CKD patients [33, 34]. In addition to this cohort study, research covering validation of immunoassays for soluble klotho showed that its concentration in CKD patients was higher than that in healthy controls [35]. These discordant results among studies on soluble alpha-klotho concentrations have been identified as a potential limitation restricting it from being used as biomarker of CKD. One possible reason for this discrepancy is the lack of a standardized assay to measure soluble klotho [41]. A previous human study evaluating three different commercial soluble klotho ELISA kits revealed poor inter- and intra-assay agreement between the ELISAs, implying that the problem may be not confined to just one ELISA manufacturer [42]. One recent study compared a commonly used commercial ELISA with immunoprecipitation-immunoblot (IP-IB) using stored serum samples of CKD patients [43]. IP-IB was strongly correlated with eGFR, whereas the commercial ELISA was not. Moreover, the IP-IB assay showed significant differences in serum klotho levels among stage 3 CKD, acute kidney injury, and end-stage renal disease groups. However, commercial ELISA failed to demonstrate differences across these groups. Therefore, this study concluded that the IP-IB assay shows better performance than the available commercial ELISA and recommended the IP-IB assay for measuring soluble klotho until a superior ELISA is developed [43]. Nevertheless, the labor-intensive and time-consuming nature of the IP-IB assay limits its applicability. Besides, this assay may be operator-dependent because it requires meticulous quality control procedures, since it involves synthetic antibody production.

Our study had some limitations. First, the expression of *klotho* genes is known to be related to age, and a decrease in soluble alpha klotho concentration has been observed in aged people [44]. However, no previous study has assessed the effect of age on soluble klotho levels in dogs. Even though serum and urinary klotho levels were not significantly different between groups divided by age, we cannot rule out the possible effect of age on klotho concentrations in our study. To verify this effect, further studies on klotho concentration with larger numbers of healthy dogs of variable ages are needed. Second, we cannot exclude discordances among klotho tissue expression, plasma klotho levels, and klotho urinary excretion, although plasma klotho was suggested to reflect membrane-bound klotho. Quantification of the membranous klotho protein on the target cells of FGF-23 would require invasive diagnostic procedures on target organs such as the kidney and parathyroid gland, which cannot be easily performed in clinical practice. Membranous klotho is cleaved and released into circulation, producing soluble klotho which is more readily accessible for measurement. However, the current methods for soluble klotho analysis may not be able to differentiate between full-length soluble klotho produced from cleavage of the membranous form [21–23] and further cleaved Kl1 and Kl2 klotho fragments. Further studies addressing the associations between different forms of klotho will improve our understanding of klotho in dogs with CKD.

## Conclusions

UrKl/Cr was decreased in dogs with advanced CKD. Furthermore, it showed a negative correlation to sSDMA, BUN, creatinine and phosphorus concentrations. These findings support the possibility that klotho is associated with CKD and its clinical consequences, including CKD-MBD, in dogs. Thus, we suggest that UrKl/Cr has the potential to serve as a biomarker, although further studies are needed to confirm its usefulness. Although serum klotho concentration was negatively correlated with sSDMA, it was not apparently related to IRIS CKD stage or other parameters known to be associated with CKD.

## Methods

### Animal selection and allocation

Client-owned dogs presenting to the veterinary teaching hospital of Chungnam National University and another referral clinic from April to October 2019 were included in this study. By the definition of CKD based on IRIS guidelines, dogs were diagnosed with CKD when they showed repeated minimally concentrated urine (urine specific gravity < 1.030) and met one of the following criteria: (1) azotemia based on serum creatinine (creatinine ≥ 1.4 mg/dL); (2) high sSDMA concentration (≥ 18 µg/dL); (3) ultrasonographic findings suggesting CKD (small, irregular kidneys or loss of corticomedullary junction). A control group comprised healthy dogs without clinical signs associated with urinary tract diseases, normal physical examination findings, normal complete blood count, and serum biochemistry profile results and urinalysis.

Twenty-seven dogs were classified into the CKD group. Their median age was 15 years (range, 5 to 19 years). Shih-tzu (*n* = 8) and Maltese terrier (*n* = 7) were the most common breeds, followed by miniature schnauzer (*n* = 2), mixed breed (*n* = 2), cocker spaniel (*n* = 1), golden retriever (*n* = 1), Japanese chin (*n* = 1), Pomeranian (*n* = 1), and Yorkshire terrier (*n* = 1). Twelve dogs were castrated males, three were intact males, nine were spayed females, and three were intact females. Dogs in the CKD group were classified into different stages according to IRIS CKD guidelines based on serum creatinine and sSDMA concentrations: (1) stage 1 (creatinine < 1.4 mg/dL and sSDMA < 18 µg/dL; *n* = 8); (2) stage 2 (creatinine 1.4 to 2.8 mg/dL or sSDMA 18 to 35 µg/dL; *n* = 8); (3) stage 3 (creatinine 2.9 to 5.0 mg/dL, sSDMA 36 to 54 µg/dL; *n* = 7); (4) stage 4 (creatinine > 5.0 mg/dL or sSDMA > 54 µg/dL; *n* = 4). Dogs were classified into different substages based on the UPC: (1) nonproteinuric (UPC < 0.2; *n* = 9); (2) borderline proteinuric (UPC: 0.2 to 0.5; *n* = 7); and (3) proteinuric (UPC > 0.5; *n* = 11). Dogs were also classified into substages based on systolic blood pressure (BP): (1) normotensive (BP < 140 mmHg; *n* = 11); (2) prehypertensive (BP: 141 to 159 mmHg; *n* = 2); (3) hypertensive (BP: 160 to 179 mmHg; *n* = 9); and (4) severely hypertensive (BP ≥ 180 mmHg; *n* = 5). Ten dogs were included in the control group; the median age was 5 years and 6 months (range, 2 to 18 years). Maltese terrier (*n* = 4) and Pomeranian (*n* = 3) were the most common breeds. Other breeds included beagle (*n* = 2) and Jindo (*n* = 1). Three dogs were castrated males, one was an intact male, four were spayed females, and two were intact females. To identify the associations between soluble alpha-klotho concentration and age, dogs in both control and CKD groups were classified into different groups based on age: (1) same or under 2 years (*n* = 3); (2) 3 to 8 years (*n* = 10); (3) 9 to 15 years (*n* = 12); and (4) 16 years or over (*n* = 12).

Dogs tentatively diagnosed with acute kidney injury or tumors in the urinary tracts were excluded. The study was conducted in animals with full consent from their owners and any treatments needed were not withheld. This study was approved by the Institutional Animal Care and Use Committee of Chungnam National University. After the sampling procedure, dogs were taken care of by their owners as usual.

### Sampling and analysis

All dogs underwent a complete physical examination, including measurement of blood pressure using a Doppler device. Blood samples were taken from the jugular vein. Within 15 minutes after collection, samples were centrifuged at 8000 rpm for 15 minutes. After measurement of blood urea nitrogen, creatinine, and inorganic phosphorus concentrations using a BS-200 analyzer (Mindray, Shenzhen, China), the remaining serum samples were frozen at − 80 °C. Urine samples were collected by either cystocentesis or catheterization after blood samples were collected. After routine urinalysis, samples were centrifuged at 3000 rpm for 20 minutes. The supernatant was frozen at − 80 °C.

Serum and urinary alpha klotho concentrations were measured using a commercially available canine-specific sandwich enzyme-linked immunosorbent assay (ELISA) kit (MyBioSource, San Diego, USA). This quantitative sandwich ELISA is based on soluble alpha klotho antibody-antigen interactions and an HRP colorimetric detection system to detect soluble alpha klotho antigen targets in samples. Samples were thawed at room temperature before klotho and sSDMA analysis. Serum SDMA level was measured with a Catalyst ONE® (IDEXX, Westbrook, USA), which is an automated analyzer that analyzes dry slides with a reactive reagent based on ELISA. BUN, creatine, and phosphorus were measured with a BS-200® (Mindray, Shenzen, China), which is an automated chemistry analyzer that uses a photometric system of 8 monochromatic lights to measure the absorbance of the reaction liquid in rotating reaction cuvettes. The urine protein-to-creatinine ratio was measured with a VetTest8008® (IDEXX, Westbrook, USA) using thawed urine samples when ELISA was performed.

### Statistical analysis

The measured parameters are provided as median value and range. The Kolmogorov–Smirnov test was used to analyze the normality of the distribution of parameters. UrKl/Cr and serum alpha klotho concentrations were not normally distributed. Therefore, UrKl/Cr and serum alpha klotho concentrations were compared between groups by a nonparametric Kruskal–Wallis test. The Mann–Whitney U-test for nonparametric data was used for confirmation of differences between groups. Spearman’s correlation coefficient was used to evaluate the relationships between variables. A *p* value of < 0.05 was considered statistically significant. A stepwise multiple regression analysis was performed with sSDMA, BUN, creatinine and phosphorus concentrations to estimate the effects of independent predictors on klotho concentrations. All analyses were performed using IBM SPSS Statistics 24 (IBM, New York, USA).

## Data Availability

The datasets generated during and/or analyzed during the current study are available from the corresponding author on reasonable request.

## Abbreviations

BUN: Blood urea nitrogen
CKD: Chronic kidney disease
CKD-MBD: Chronic kidney disease-mineral bone disorder
ELISA: Enzyme-linked immunosorbent assay
FGF: Fibroblast growth factor
eGFR: Estimated glomerular filtration rate
GFR: Glomerular filtration rate
IP-IB: Immunoprecipitation-immunoblot
IRIS: International Renal Interest Society
RSHP: Renal secondary hyperparathyroidism
sSDMA: Serum symmetric dimethylarginine
UrKl/Cr: Urine klotho-to-creatinine ratio
UPC: Urine protein-to-creatinine ratio
